# Isolation and Characterization of Microsatellite Loci for *Hibiscus aridicola* (Malvaceae), an Endangered Plant Endemic to the Dry-Hot Valleys of Jinsha River in Southwest China

**DOI:** 10.3390/ijms12095698

**Published:** 2011-09-05

**Authors:** Le Zhang, Weibang Sun, Zhonglang Wang, Kaiyun Guan, Junbo Yang

**Affiliations:** 1 Kunming Botanical Garden, Kunming Institute of Botany, Chinese Academy of Sciences, Kunming 650204, China; E-Mails: zhangle@mail.kib.ac.cn (L.Z.); wang@mail.kib.ac.cn (Z.W.); guanky@mail.kib.ac.cn (K.G.); 2 Graduate University of Chinese Academy of Sciences, Beijing 100049, China; 3 Key Laboratory of Plant Biodiversity and Biogeography, Kunming Institute of Botany, Chinese Academy of Sciences, Kunming 650204, China; E-Mail: jbyang@mail.kib.ac.cn

**Keywords:** *Hibiscus aridicola*, endangered plant, SSR markers, population structure, population genetics

## Abstract

*Hibiscus aridicola* (Malvaceae) is an endangered ornamental shrub endemic to the dry-hot valleys of Jinsha River in southwest China. Only four natural populations of *H. aridicola* exist in the wild according to our field investigation. It can be inferred that *H. aridicola* is facing a very high risk of extinction in the wild and an urgent conservation strategy is required. By using a modified biotin-streptavidin capture method, a total of 40 microsatellite markers were developed and characterized in *H. aridicola* for the first time. Polymorphisms were evaluated in 39 individuals from four natural populations. Fifteen of the markers showed polymorphisms with two to six alleles per locus; the observed heterozygosity ranged from 0.19 to 0.72. These microsatellite loci would be useful tools for population genetics studies on *H. aridicola* and other con-generic species which are important to the conservation and development of endangered species.

## 1. Introduction

Genus *Hibiscus* (Malvaceae) includes at least 250 species distributed in tropical and subtropical regions [1]. *H. aridicola* is endemic to the dry and hot valleys of the Jinsha River (upper reaches of the Yangtse River) in Southwest China. It is an ornamental deciduous shrub with large flowers that vary from white to whitish purple. Although the species was described in 1927 [2], and literature or relative research references are limited. *H. aridicola* was widely distributed along the valleys of Jinsha River from 1300 m to 2100 m during the 1950s [2–4]. However, it currently has been evaluated as Endangered [EN B2ab (ii)] (following IUCN Red List Categories and Criteria) with less than five locations and a continuing decline of area of occupancy [5]. Our recent field surveys have further confirmed the threatened status of the species and only four natural populations of *H. aridicola* exist in the wild. Our surveys have also discovered that habitats of *H. aridicola* have been greatly degraded and the populations are fragmented and isolated from each other. Population genetic studies have predicted that fragmentation will lead to a loss of genetic diversity due to inbreeding, population isolation and restricted gene flow and small effective population sizes and that may lead to a decline in fitness or even, ultimately, extinction [6,7]. As an endangered and endemic ornamental shrub, *H. aridicola* is facing a very high risk of extinction in the wild, and its effective and long-term conservation is urgently needed. In our study, 40 microsatellite markers were developed and characterized to investigate the genetic diversity and population structure using the fast isolation by Amplified Fragment Length Polymorphism (AFLP) of sequences containing repeats (FIASCO) [8].

## 2. Results and Discussion

A total of 441 positive clones were captured, among these 232 clones (53%) were found to contain simple sequence repeat (SSR). Finally, 84 sequences contained SSR loci were selected for primer design. 40 microsatellite loci successfully amplified in *H. aridicola*. We tested the degree of polymorphism in four populations of *H. aridicola* for 40 microsatellite loci and 15 out of them are polymorphic amplification, other 25 microsatellite loci were monomorphic by the result of polyacrylamide gel (Table 1).

The number of alleles ranged from two to six in 39 individuals of the species sampled from the four extant natural populations. Value for *H*_O_ and *H*_E_ ranged from 0.19 to 0.72 (mean *H*_O_ = 0.52) and from 0.20 to 1.00 (mean *H*_E_ = 0.62), respectively (Table 2).

These microsatellite markers developed in our study will be a useful tool for further studies of conservation genetics, and will help us understand the genetic structure of *H. aridicola*, so as to make effective conservation strategy for this endangered plant.

## 3. Experimental Section

Total genomic DNA of *H. aridicola* was extracted from dry leaf tissue of three different individuals using a modified 4 × CTAB method [9]. A microsatellite enriched library was conducted by using a modified biotin-streptavidin capture method. Firstly, the genomic DNA (about 800–1000 ng) was completely digested with *Mse*I restriction enzyme (NEB) into the sequences between 200 bp–800 bp, then the digested fragments were ligated to *Mse*I AFLP adaptor following by amplification with adaptor-specific primers (5′-GAT GAG TCC TGA GTA). After a PCR the products were hybridized with biotinylated probes (AG)_15_, (AC)_15_ and (AAG)_10_ respectively [8]. The PCR products were ligated into PGEM-T vector (Promega) after purified, and transferred into *E. coli* strain DH5α (Tiangen). The proportion of products and PGEM-T vector in the total volume should be controlled between 3:1 and 5:1. Positive clones were picked out and tested using (AG)_10_/(AC)_10_/(AAG)_7_ primers and vector primers SP6/T7 respectively to select appropriate fragments which contained SSR. The positive clones were captured for sequencing with an ABI PRISM 3730XL SEQUENCER. Sequences contained simple sequence repeat were selected for primer design using Primer Premier 5.0 program [10].

The designed Primer pairs were tested in 39 wild *H. aridicola* samples from four populations in Southeast China. Herbarium voucher deposited in Kunming Institute of Botany, Chinese Academy of Sciences (code ZL0017-0019, ZL0108- ZL 0120). The PCR amplification was carried out in a total volume of 10 μL reaction containing 5 μL 2 × Taq PCR MasterMix (Tiangen; 0.1 U Taq Polymerase/μL, 0.5 mM dNTP each, 20 mM Tris-HCl (Ph8.3), 100 mM KCl, 3 mM MgCl_2_), 0.5 μL of each primer and 0.5 μL Genomic DNA. Amplification was carried out in thermocycler (veriti 96 well thermal cycler) with a cycling profile 95 °C for 4 min then 35 cycles of 45 s at 94 °C, 45 s at the specific annealing temperature, 45 s at 72 °C and a final extension step of 8 min at 72 °C. The amplification products were separated on 8% denaturing polyacrylamide gels and visualized by silver staining with a 100 bp extended DNA ladder (Fermentas) as a size standard.

The data was analyzed by GENEPOP 4.0 [11], which included test of observed heterozygosity (*H*_O_) and expected heterozygosity (*H*_E_) for the 15 polymorphic microsatellite loci.

## 4. Conclusions

In summary, 40 microsatellite markers have been specifically developed for *H. aricicola* in this study. The high discriminatory power of 15 polymorphic loci suggests that they should be suitable for the fine-scale analysis and survey of population structure in scarce populations of the endangered *H. aridicola*. These developed and characterized SSR markers for *H. aridicola* would also be useful for exploring genetic diversity and genetic structure of other species in *Hibiscus*.

## Figures and Tables

**Table 1:** Primer sequences and characteristics of 40 microsatellite loci successfully amplified in 39 *Hibiscus aridicola* individuals.

Locus	Primer Sequence (5′-3′)	Repeat motif	NA	Size (bp)	*T*_a_ (°C)	Gene Bank Accession No.
HA-1	F: TTGAACATAAACAAGCGG R: AAAACAAGTTGGGGAGG	(GT)_14_	2	180–200	56	JN167557
HA-2	F: CTGAATGCCAGAATGACT R: CAGGCGAAAGAGGAAGAT	(AG)_21_	5	410–445	60	JN167558
HA-3	F: ATCATTATCATCTTCGTTTC R: AAGGGACCAAAGTCTCAA	(CT)_15_	2	141–154	59	JN167559
HA-4	F: CACCAAATCCTGGAGAAG R: GCAAACGAGAATAATCAAAA	(AC)_11_	2	137–150	60	JN167560
HA-5	F: GCGTGGATGTTCTTTCTT R: TCGAACCCTATGGATGTA	(GA)_8_	3	155–175	61	JN167561
HA-6	F: GAACAAGCCTGTCACTAA R: CACAAACCGATTTACGAT	(CT)_18_	2	130–178	60	JN167562
HA-7	F: CAGCAGTTAGAGCAGGAGGT R: TTCGGACATGAGTATGGGAT	(TG)_8_	3	208–215	59	JN167563
HA-8	F: CACTTCCACGAAGCTCTTAC R: GGAGATAAACAGAAAAGGGTA	(CTTCT)_3_	2	230–242	59	JN167564
HA-9	F: TATGGGTTTAGTGCCTGTAT R: TAGGTTGCTTGAATCTTTTC	(AC)_9_	2	322–341	59	JN167565
HA-10	F: CCCAAACCTCTATCATCT R: ATATCCCTTAGTTCTGCT	(GT)_11_	2	197–204	59	JN167566
HA-11	F: CACCAAATCCTGGAGAAGTA R: GGCAAACGAGAATAATCAAA	(AC)_9_	3	98–109	58	JN167567
HA-12	F: AAGGAGAAGCCAAGGTGAAA R: GACAAACCCACATACAGGAA	(GAA)_5_	2	119–132	60	JN167568
HA-13	F: ACTTTTATCGTATAGACCAG R: GAACACCTTTATTTCAGTGT	(CTT)_15_	2	110–118	59	JN167569
HA-14	F: GAAATGGCAAGGTTTTAGAT R: CTCAACTTTTGTGATGTGGC	(GAA)_10_	3	144–153	59	JN167570
HA-15	F: CAGCCACAATCCTCCTAACT R: GAAGGGTAACTTGTTTCACG	(TG)_11_	3	313–345	60	JN167571
HA-16	F: TTGAGATTTGACCTGGAA R: ACATTGGCGAAGATACAC	(CT)_20_	1	237	57	JN167572
HA-17	F: TATTTCCCTGTCCCTGTT R: GACCTTTTCGTCTTTTGG	(CT)_14_	1	74	54	JN167573
HA-18	F: CACCCAAGCATGATAAAA R: AGAATGAAAGAAAATGGC	(AC)_14_	1	164	58	JN167574
HA-19	F: ACCACCAGAAAGCAAACA R: GATGACTAATGGGAAAGAA	(CT)_12_	1	122	58	JN167575
HA-20-	F: TCGTGATGGGAACAGATA R: TGAAATACTCATGGGAATG	(GA)_13_	1	141	57	JN167576
HA-21	F: AGAAAATCCCAATCTCAA R: CTAGCCAGAAACAACGAG	(CT)_16_	1	201	61	JN167577
HA-22	F: ACTGGTAACATCCCTGAC R: GAAACTGCTGGAAATCTA	(AC)_9_	1	107	60	JN167578
HA-23	F: AGCATCCGATCCTTATCT R: TATCAGCGACTCCTCCAC	(CT)_16_	1	158	59	JN167579
HA-24	F: AGTCATCGGAGAAATAGAG R: ATAACCAAGGAGGAAACA	(CAT)_9_	1	442	58	JN167580
HA-25	F: AAACTGCGAAATCCTCAT R: AGTAAACACTGCCTCCAT	(CT)_16_(CA)_8_	1	100	60	JN167581
HA-26	F: CCTCCGTGGTAACTCCTT R: TGATGAAATATGGCTTGG	(CA)_11_	1	137	59	JN167582
HA-27	F: TGAATTTCTTTTCTTCCTTTAC R: CAACTATCATCTTGTCGTGC	(TG)_10_	1	207	58	JN167583
HA-28	F: ATAGATGAACCAGGAAAT R: CTGAAGATAAAGAAAGCA	(AC)_10_	1	102	53	JN167584
HA-29	F: ATACGACAGATGCGGAAGTG R: TTAGTTACGGGAACCGAAGG	(GAA)_3_	1	196	60	JN167585
HA-30	F: TTGCTCACTTGAAAACATTA R: GAAAACGACACGATCACTCT	(AC)_10_	1	72	58	JN167586
HA-31	F: GGAAAGTGGCTGACTGGTAG R: CGACATCGGTGAGGTTGGTT	(AC)_7_	1	296	60	JN167587
HA-32	F: ACGGAAATGCTCAAACCCTC R: AAATGATTACCGCCGACAAC	(CTT)_3_	1	121	58	JN167588
HA-33	F: GCTCAGGTAAACCCATAA R: GCTCGTCGTACATACACTT	(CT)_7_	1	300	55	JN167589
HA-34	F: TACTGTCCAATGAATGCCTT R: AACCTGAACTATAAAGTAAACTGC	(GT)_14_	1	155	60	JN167590
HA-35	F: GTATGTTGCTATCCCCTAT R: CAAACCAAACAACACTAA	(TG)_8_	1	139	60	JN167591
HA-36	F: GAAAGGAATTGTACGTGGCA R: TATGGCTTGGGATTGGTTTT	(CA)_8_	1	264	60	JN167592
HA-37	F: TAAGATGGTATTGGAAGGG R: AGGGAGCATAAAAGTGGT	(GT)_11_	1	345	60	JN167593
HA-38	F: ATCATCGGCAGCGACTAG R: CAGCAAGGACATCAGGGT	(GA)_13_	1	203	59	JN167594
HA-39	F: TCCAAGATACTGCCATAC R: GGTTCTACAGGTACATGC	(AC)_14_	1	108	60	JN167595
HA-40	F: GAAGAGCGACAGAAAATG R: GGAAAATAAACAAGGGTAAA	(AC)_14_	1	103	59	JN167596

*T*_a_, PCR annealing temperature; *N*_A_, number of alleles; Size range, size of alleles.

**Table 2:** Results of 15 polymorphic primers screening in four populations of *H. aridicola*.

	Population 1 (*N* = 10)			Population 2 (*N* = 10)			Population 3 (*N* = 10)			Population 4 (*N* = 9)		
	
Locus	*N*_A_	*H*_O_	*H*_E_	*N*_A_	*H*_O_	*H*_E_	*N*_A_	*H*_O_	*H*_E_	*N*_A_	*H*_O_	*H*_E_
HA-1	2	0.51	0.70	2	0.51	0.80	2	0.48	0.70	2	0.47	0.67
HA-2	5	0.72	1.00	4	0.49	0.60	3	0.59	0.60	4	0.66	0.78
HA-3	2	0.19	0.20	2	0.48	0.10	2	0.47	0.40	2	0.52	0.00
HA-4	2	0.51	0.80	2	0.39	0.50	2	0.48	0.70	2	0.53	1.00
HA-5	3	0.62	0.70	3	0.57	0.40	3	0.54	0.70	3	0.46	0.56
HA-6	2	0.48	0.70	2	0.39	0.50	2	0.48	0.70	2	0.53	1.00
HA-7	3	0.47	0.50	2	0.44	0.60	3	0.56	0.90	2	0.37	0.44
HA-8	2	0.52	0.70	3	0.57	0.60	3	0.65	0.70	2	0.53	0.78
HA-9	2	0.44	0.60	2	0.52	0.70	2	0.44	0.60	2	0.50	0.78
HA-10	2	0.39	0.50	2	0.44	0.60	2	0.39	0.50	2	0.37	0.44
HA-11	3	0.51	0.70	2	0.51	0.60	3	0.62	0.70	3	0.69	0.78
HA-12	2	0.44	0.60	3	0.66	1.00	3	0.65	0.90	3	0.60	0.89
HA-13	2	0.39	0.30	3	0.57	0.60	3	0.42	0.30	3	0.65	0.56
HA-14	3	0.42	0.40	4	0.57	0.30	3	0.43	0.20	3	0.57	0.44
HA-15	3	0.53	0.70	3	0.61	0.70	3	0.68	1.00	3	0.68	0.89

*N* = population samples size; *N*_A_, number of alleles; *H*_O_, observed heterozygosity; *H*_E_, expected heterozygosity; Population 1 (27°15′N, 102°53′E); Population 2 (27°44′N, 100°24′E); Population 3 (27°45′N, 100°18′E); Population 4 (27°29′N, 100°09′E).
